# Human phosphodiesterase 4D7 (PDE4D7) expression is increased in *TMPRSS2-ERG*-positive primary prostate cancer and independently adds to a reduced risk of post-surgical disease progression

**DOI:** 10.1038/bjc.2015.335

**Published:** 2015-11-17

**Authors:** R Böttcher, D J P Henderson, K Dulla, D van Strijp, L F Waanders, G Tevz, M L Lehman, D Merkle, G J L H van Leenders, G S Baillie, G Jenster, M D Houslay, R Hoffmann

**Affiliations:** 1 Department of Urology, Erasmus Medical Center, Rotterdam 3000 CA, The Netherlands; 2 Institute of Cardiovascular and Medical Science, University of Glasgow, Glasgow G12 8TA, Scotland; 3 Departments of Oncology Solutions and Precision Diagnostics, Philips Research Europe, Eindhoven 5656 AE, The Netherlands; 4 Australian Prostate Cancer Research Centre—Institute of Health and Biomedical Innovation, University of Technology, and Translational Research Institute, Brisbane, Queensland 4102, Australia; 5 Department of Pathology, Erasmus Medical Center, Rotterdam 3000 CA, The Netherlands; 6 Institute of Pharmaceutical Science, King's College London, London WC2R 2LS, UK

**Keywords:** *TMPRSS2-ERG* gene rearrangement, androgen receptor, PKA, cAMP, phosphodiesterase, androgen-independence, prostate cancer progression

## Abstract

**Background:**

There is an acute need to uncover biomarkers that reflect the molecular pathologies, underpinning prostate cancer progression and poor patient outcome. We have previously demonstrated that in prostate cancer cell lines PDE4D7 is downregulated in advanced cases of the disease. To investigate further the prognostic power of PDE4D7 expression during prostate cancer progression and assess how downregulation of this PDE isoform may affect disease outcome, we have examined PDE4D7 expression in physiologically relevant primary human samples.

**Methods:**

About 1405 patient samples across 8 publically available qPCR, Affymetrix Exon 1.0 ST arrays and RNA sequencing data sets were screened for PDE4D7 expression. The *TMPRSS2-ERG* gene rearrangement status of patient samples was determined by transformation of the exon array and RNA seq expression data to robust *z*-scores followed by the application of a threshold >3 to define a positive *TMPRSS2-ERG* gene fusion event in a tumour sample.

**Results:**

We demonstrate that PDE4D7 expression positively correlates with primary tumour development. We also show a positive association with the highly prostate cancer-specific gene rearrangement between TMPRSS2 and the ETS transcription factor family member ERG. In addition, we find that in primary *TMPRSS2-ERG*-positive tumours PDE4D7 expression is significantly positively correlated with low-grade disease and a reduced likelihood of progression after primary treatment. Conversely, PDE4D7 transcript levels become significantly decreased in castration resistant prostate cancer (CRPC).

**Conclusions:**

We further characterise and add physiological relevance to PDE4D7 as a novel marker that is associated with the development and progression of prostate tumours. We propose that the assessment of PDE4D7 levels may provide a novel, independent predictor of post-surgical disease progression.

Prostate cancer is the most commonly occurring non-skin malignancy in men, with an estimated 900 000 new cases diagnosed world-wide in 2013 (Ferlay *et al*, 2015). However, reactive clinical intervention after routine diagnosis often leads to significant over-treatment of non-aggressive tumours. This has severe negative impacts on both patient quality of life and the medical resources of healthcare institutions (Andriole *et al*, 2009; Schröder *et al*, 2009). Therefore, the characterisation of new biomarkers and methods of clinical assessment is of significant importance when assessing the need for different forms of clinical intervention.

Previous studies have shown that signalling pathways mediated by the second messenger cAMP have various roles in the development and progression of prostate cancer (Merkle and Hoffmann, 2011). Cyclic nucleotide phosphodiesterases (PDEs) (Conti and Beavo, 2007; Maurice *et al*, 2014) provide the sole means of degrading cAMP and cGMP in cells, and are pivotally placed to regulate cAMP signalling by virtue of their intracellular location and post-translational modification (Lugnier, 2006; Houslay, 2010). Each of the 11 PDE genes encode for a series of isoform variants, thereby greatly increasing the diversity of unique regulatory mechanisms, intracellular targeting and kinetic properties, which define functionally independent and unique signalling roles within the cell (Houslay *et al*, 2007; Houslay, 2010; Francis *et al*, 2011). This diversity underpins a paradigm of compartmentalised, temporally gated cyclic nucleotide signalling. Due to the complexity of these orchestrated signalling events, any change in PDE isoform expression or regulation can functionally contribute to disease onset (Lee *et al*, 2012; Michot *et al*, 2012; Apuhan *et al*, 2013; Kaname *et al*, 2014; Yoon *et al*, 2014). The molecular characterisation of these changes can be expected to provide means for the development of novel therapeutics and diagnostics (Houslay *et al*, 2005; Houslay, 2010).

Members of the PDE4D subfamily have been implicated as underpinning the molecular pathology of various diseases including prostate cancer (Rahrmann *et al*, 2009; Henderson *et al*, 2014), stroke (Gretarsdottir *et al*, 2003), acrodysostosis (Kaname *et al*, 2014) and COPD (Yoon *et al*, 2014). The *PDE4D* gene encodes a cohort of isoforms that are classified as long, short and super-short. Long isoforms possess two conserved regulatory domains, called UCR1 and UCR2, which allow long isoforms to be phosphorylated and activated by PKA (3′,5′ cAMP-dependent protein kinase) after cAMP elevation in cells (Hoffmann *et al*, 1998), as well as being functionally regulated through phosphorylation by activated forms of ERK, MK2 and AMPK (MacKenzie *et al*, 2011; Sheppard *et al*, 2014).

PDE4D7 is a long isoform member of this subfamily (Wang *et al*, 2003). We have demonstrated that PDE4D7 exhibits a specific pattern of intracellular localisation in prostate cancer cells, where it is functionally targeted to the sub-plasma membrane compartment (Henderson *et al*, 2014). Spatially constrained PDE4D7 appears to perform a pivotal role in these cells by desensitising sub-plasma membrane-localised cAMP signalling (Henderson *et al*, 2014), as well as providing a node for cross-talk with signalling pathways that elicit the activation of Erk, MK2 and AMPK (Hoffmann *et al*, 1999; Baillie *et al*, 2000; MacKenzie *et al*, 2011; Sheppard *et al*, 2014). PDE4D7 activity is also regulated by PKA phosphorylation within its unique N-terminal region (Byrne *et al*, 2015). Interestingly, susceptibility markers for ischaemic stroke also map to the region of Chr5q12, where *PDE4D7* and the androgen-regulated *PART1* exons are located (Gretarsdottir *et al*, 2003).

We have previously demonstrated that PDE4D7 is highly expressed in androgen-responsive prostate cancer cell lines and xenografts, while being downregulated in castration resistant samples (Henderson *et al*, 2014). Indeed, the ectopic overexpression of PDE4D7 in castration resistant prostate cancer (CRPC) cell lines reduced cellular proliferation, while specific knockdown of the PDE isoform in androgen-sensitive cells lead to an increase in cellular proliferation, indicating a functional role of PDE4D7 downregulation during the progression to CRPC growth. Here, we set out to assess whether the changes in PDE4D7 expression we observed in model systems have clinical relevance. To do this, we analysed 1405 tumour samples sourced from 8 independent patient cohorts that were enroled at different clinical centres (Supplementary Table 1). Our analyses of clinical samples highlight an increase in PDE4D7 expression during initial tumorigenesis and further support our contention that PDE4D7 levels then fall profoundly in CRPC, suggesting that PDE4D7 transcripts may provide a potentially useful biomarker and therapeutic target.

## Materials and Methods

### Human tissue samples

**Figure 1:**
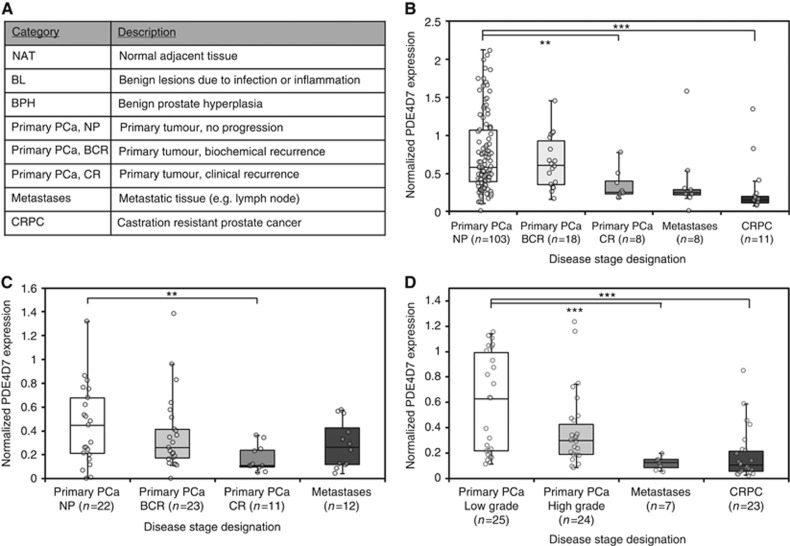
**Expression of PDE4D7 splice variant in primary, metastatic and castration resistant cancerous prostate tissues.** Box-and-Whisker plots of the normalised PDE4D7 transcript expression in various prostate cancer tissues. For all data sets, and all *P*-values and AUCs see Supplementary Table 5. (**A**) Disease stage annotation for this study. (**B**) Data: Taylor *et al*, 2010; *P*-values of group comparison for difference of mean PDE4D7 expression: (Primary PCa, NP) *vs* (Primary PCa, BCRandCR), *P*=7.2E−02; (Primary PCa, NP&BCR) *vs* (Primary PCa, CR), *P*=5.90E−03; (Primary PCa, all) *vs* (Metastases), *P*=1.60E−01; (Primary PCa, all) *vs* (CRPC), *P*=5.8E−04; (**C**) Data: Boormans *et al*, 2013; Böttcher *et al*, 2015; *P*-values of group comparison for difference of mean PDE4D7 expression: (Primary PCa, NP) *vs* (Primary PCa, BCR&CR), *P*=6.50E−02; (Primary PCa, NP) *vs* (Primary PCa, CR), *P*=1.30E−03; (Primary PCa, all) *vs* (Metastases), *P*=1.1E−01; (**D**) Data: J Schalken, Personal Communication; *P*-values of group comparison for difference of mean PDE4D7 expression: (Primary PCa, low grade) *vs* (Primary PCa, high grade), *P*=2.0E−01; (Primary PCa, all grades) *vs* (Metastases), *P*=4.60E−04; (Primary PCa, all grades) *vs* (CRPC), *P*=1.90E−05. **P<0.01 and ***P<0.001.

Human tissues samples were obtained under local laws and regulation to obtain and handle patient material for research purposes. Sample descriptions are depicted in Figure 1A.

### Molecular biology (RNA extraction, cDNA synthesis and primer design)

If not otherwise indicated RNA isolation, cDNA conversion and Real-Time PCR were performed using RNeasy Kit (QIAGEN GmbH, Hilden, Germany, 74004), iScript cDNA synthesis kit (Bio-Rad Inc, Hercules, CA, USA, 170–8890), GeneAmp Fast PCR Master Mix (Applied Biosystems Inc, Foster City, CA, USA, 4362070) respectively, according to the manufacturer's instruction. Real-Time PCR probe and primer sets were developed by targeting isoform-specific intron-spanning regions of genetic code (Supplementary Table 3).

### Quantitative RT–PCR (qRT–PCR)

To enable the comparison of qPCR data across different experiments, we normalised the Ct value for PDE4D7 against the mean of the Ct values for the reference genes (Supplementary Table 3) to generate a normalised PDE4D7 expression value.

We use the following formula to normalise the raw Ct values:


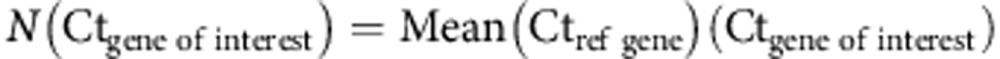


Where N(Ct_gene of interest_)=is normalised gene expression value for a gene of interest; where Mean(Ct_ref gene_) is the arithmetic mean of the PCR Cq values of the selected combination of reference genes; where (Ct_gene of interest_) is the PCR Cq value of the gene of interest.

Note: in case DNA microarray or RNA seq technologies was used to measure PDE4D7 expression, the qPCR Ct value was replaced by a normalised measurement of the respective technology, for example, an robust multi-array average (RMA) normalised gene expression value for DNA microarrays, or a TPM (transcript per million) normalised gene expression value for RNA sequencing.

### Analysis of affymetrix human exon arrays

Raw CEL files were downloaded from Gene Expression Omnibus for the publically available data sets (Supplementary Table 1). Data processing and RMA normalisation were performed using the aroma.affymetrix R-package (Affymetrix Inc, Santa Clara, CA, USA; Purdom *et al*, 2008) and transcript isoform expression was measured by averaging log2-transformed intensity values of the following isoform-specific probe sets: PDE4D7 (2858406, 2858407 and 2858408); Note: for data set Erho *et al* (2013) (Supplementary Table 1) only probe set 2858408 was used in the analysis as probe sets 2858406 and 2858407 showed relatively limited signal intensities compared with probe set 2858408.

### RNA seq data analysis

RNA seq data of 193 prostate cancer clinical samples (36 normal, 157 tumour) was downloaded from The Cancer Genome Atlas (TCGA) Data Portal (4 September 2013) and the expression value of genes and isoforms (TPM-transcript per million) was estimated as previously described (Li *et al*, 2010).

Positive *TMPRSS2-ERG* fusion status was estimated in general by transformation to robust *z*-scores. Positive TMPRSS2-ERG fusion status was estimated by transformation to robust *z*-scores, utilising robust statistical measures, namely median and median absolute deviation, to replace mean and s.d., which are sensitive to outliers. Thus, log2-transformed expression values were converted by *z*-score=(expression−median(expression))/(MAD(expression)), and a threshold of >3 was applied to define samples with positive fusion events. Subsequently, a threshold of >3 was applied to define samples with positive fusion events. For the Erho *et al* (2013) data set, we applied a supervised clustering algorithm (Partitioning Around Medoids) to assign prostate cancer samples in one of the two clusters (high ERG or low ERG) based on the log2-transformed expression values of ERG. High ERG expression was subsequently assumed as representative for the presence of a positive *TMPRSS2-ERG* fusion event.

To assess whether any evidence of ERG binding in the genomic region of PDE4D could be observed, we utilised public ChIP-seq data (GSE14092) from the VCaP prostate cancer cell line after liftOver (https://genome.ucsc.edu/cgi-bin/hgLiftOver) to hg19 and found 43 peaks overlapping PDE4D when including 50-kb flanking regions. One of these peaks overlapped the PDE4D7 promoter region, while another was located in close proximity (<200 bases distance), which may hint towards an involvement of ERG binding in regulation of PDE4D7 expression.

### Statistical data analysis

For ROC analysis, calculation of AUC under the ROC, ROC *P*-values and Box-and-Whisker plots the statistical software package MedCalc (MedCalc Software BVBA, Ostend, Belgium) was used. *P*-values for differences of mean expression were calculated by using Wilcoxon–Mann–Whitney testing unless mentioned otherwise.

Kaplan–Meier Survival curves have been generated by the medical statistical software package MedCalc based on the time to event for those patients who experienced the respective event (e.g., biochemical recurrence (BCR) or clinical recurrence (CR) of disease after surgery) and for those patients who did not suffer from the event at the time of follow-up (censored data). Further, to segregate the analysed patient cohort into two survival groups we determined a cut-off of PDE4D7 expression from a ROC curve analysis. The respective cut-off was objectively determined from the ROC curve at the unique point in the curve, where the sum of sensitivity and specificity reached a maximum.

## Results

We have recently provided evidence, suggesting that PDE4D7 may play an important role in regulating cAMP signalling during prostate cancer progression (Henderson *et al*, 2014). To further explore this finding, we have evaluated the expression of PDE4D7 in a total of eight clinically relevant patient data sets. These data sets comprised a total of 1405 patient samples stratified into 8 sample categories listed in Figure 1A. Three different technology platforms were also leveraged to ensure reproducibility and significance of the gene expression data for PDE4D7, namely: (1) qPCR; (2) Affymetrix Human Exon Array 1.0 ST; (3) RNA seq (see Supplementary Table 1). More details of the data sets used within this study can be found in Supplementary Tables 1 and 2.

### PDE4D7 expression correlates with primary localised prostate tumours and is significantly downregulated in CRPC

Our previous investigation in cell lines and xenograft material found that PDE4D7 was differentially expressed between androgen sensitive/responsive and CRPC cells (Henderson *et al*, 2014). To assess if this finding is physiologically relevant, we thought it prudent to examine PDE4D7 transcript expression in primary patient samples. We selected three prostate cancer exon array data sets (Taylor *et al*, 2010; Boormans *et al*, 2013; Böttcher *et al*, 2015; J Schalken, Radboud Uinversity Nijmegen Medical Center, Nijmegen, The Netherlands, Personal Communication; Supplementary Table 1) and analysed a range of primary prostate cancer samples including tissues collected from patients who developed biochemical or clinical tumour progression after primary treatment, as well as metastases and CRPC (Figure 1B–D; Supplementary Table 5). We observed a striking downregulation in PDE4D7 expression between primary prostate cancer without tumour progression (Primary PCa, NP) and primary prostate cancer tissue with either progression to BCR (Primary PCa, BCR) or CR (Primary PCa, CR). The ROC analysis for the group-wise comparisons revealed AUCs are between 0.61 and 0.82 (Supplementary Table 5). In line with our previous findings, the most significant downregulation was observed between tissues representing primary prostate cancer *vs* CRPC (data sets Taylor *et al*, 2010 and J Schalken, Personal Communication; *P*-values for differential PDE4D7 expression 5.80E−04, and 1.90E−05, respectively; AUCs for PDE4D7 ROC analysis 0.82, 95% CI 0.73–0.88 and 0.81, 95% CI 0.71–0.90, respectively; Supplementary Table 5). In contrast to the comparison between primary tumours and CRPC, a differential expression of PDE4D7 between primary prostate cancer and metastatic tissue could not be confirmed in the data set from Taylor *et al* (2010) (*P*=1.60E−01; AUC=0.65; 95% CI 0.55–0.74) nor in Boormans *et al* (2013); Böttcher *et al* (2015) (*P*=1.10E−01; AUC=0.67; 0.49–0.82); however, in the data set produced by J Schalken, Personal Communication the expression difference was significant (*P*=4.6E−04) with a very large AUC (0.91; 95% CI 0.81–0.97). Overall this data confirms our original observation made in *in vitro* models of prostate cancer; PDE4D7 is significantly downregulated in aggressive and advanced forms of prostate cancer.

### PDE4D7 expression is upregulated in localised primary prostate tumours and correlates with *TMPRSS2-ERG* gene fusion

**Figure 2:**
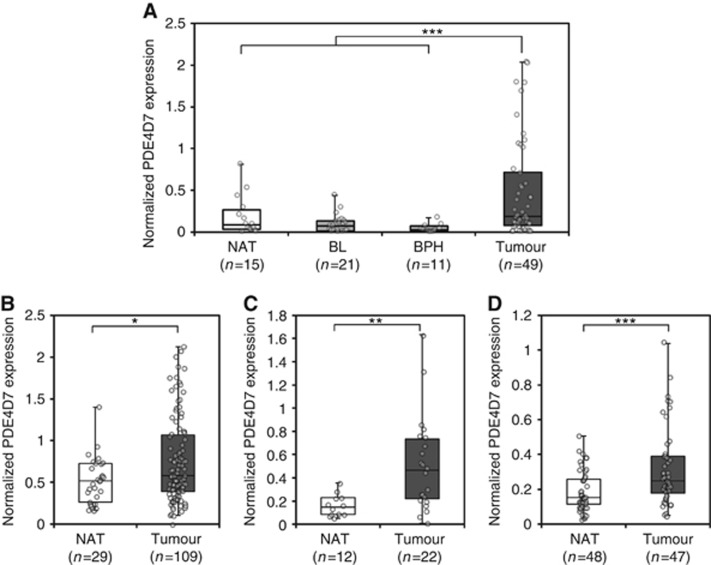
**Expression of PDE4D7 splice variant in normal, benign *vs* cancerous prostate tissues.** Box-and-Whisker plots of the normalised PDE4D7 transcript expression in various prostate cancer tissues. For all data sets and all *P*-values see Supplementary Table 6. (**A**) Data: Origene; *P*-values of group comparison for difference of mean PDE4D7 expression: (NAT) *vs* (Primary PCa), *P*=6.86E−02; (NAT&BL&BPH) *vs* (Primary PCa), *P*=1.31E−04; (BL&BPH) *vs* (Primary PCa), *P*=4.0E−04; (BPH) *vs* (Primary PCa), *P*=3.2E−02; (**B**) Data: Taylor *et al*, 2010; *P*-values of group comparison for difference of mean PDE4D7 expression: (NAT) *vs* (Primary PCa), *P*=3.30E−02; (**C**) Data: Boormans *et al*, 2013; Böttcher *et al*, 2015; *P*-values of group comparison for difference of mean PDE4D7 expression: (NAT) *vs* (Primary PCa), *P*=3.50E−03; (**D**) Data: Brase *et al*, 2011; *P*-values of group comparison for difference of mean PDE4D7 expression: (NAT) *vs* (Primary PCa), *P*=1.00E−03. *P<0.05, **P<0.01 and ***P<0.001.

To assess the significance of PDE4D7 expression within the context of the normal prostate epithelia, we extended the exon array analysis to include patient tissue taken from areas adjacent to prostate tumours (NAT). We examined 850 patient samples across seven independent data sets (Supplementary Tables 1 and 2). Interestingly, we observed a significant upregulation of PDE4D7 in primary prostate cancer *vs* NAT (Figure 2A–D; Supplementary Table 6). This suggests that PDE4D7 upregulation in prostate tissue may be involved with initial tumorigenesis.

**Figure 3:**
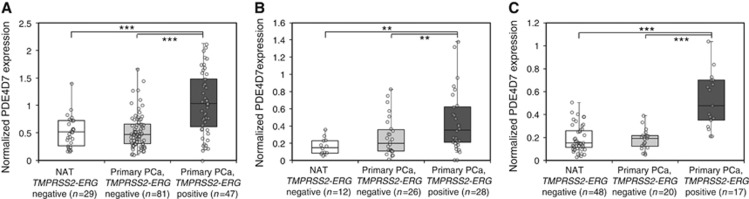
**Correlation of PDE4D7 expression in normal and cancerous human prostate tissues to TMPRSS2-ERG gene fusion status.** (**A**) Box-and-Whisker plots of the normalised PDE4D7 transcript expression in various prostate cancer tissues. For all data sets, and all *P*-values see Supplementary Table 7. Positive *TMPRSS2-ERG* fusion status was estimated by transformation to robust *z*-scores (Materials and Methods). Subsequently, a threshold of >3 was applied to define samples with positive fusion events. Samples were divided into three different groups: (1) normal adjacent tissue without *TMPRSS2-ERG* fusion events (NAT *TMPRSS2-ERG* negative); (2) prostate tumour tissue without *TMPRSS2-ERG* fusion events (Primary PCa, *TMPRSS2-ERG* negative), and (3) prostate tumour tissue with TMPRSS2-ERG fusion events (Primary PCa, *TMPRSS2-ERG* positive). (**A**) Data: Taylor *et al*, 2010; *P*-values of group comparison for difference of mean PDE4D7 expression: (NAT *TMPRSS2-ERG* negative) *vs* (Primary PCa, *TMPRSS2-ERG* negative), *P*=9.00E−01; (NAT *TMPRSS2-ERG* negative) *vs* (Primary PCa, *TMPRSS2-ERG* positive), *P*=1.10E−05; (Primary PCa, *TMPRSS2-ERG* negative) *vs* (Primary PCa, *TMPRSS2-ERG* positive), *P*=3.33E−08. (**B**) Data: Boormans *et al*, 2013; Böttcher *et al*, 2015; *P*-values of group comparison for difference of mean PDE4D7 expression: (NAT *TMPRSS2-ERG* negative) *vs* (Primary PCa, *TMPRSS2-ERG* negative), *P*=5.90E−01; (NAT *TMPRSS2-ERG* negative) *vs* (Primary PCa, *TMPRSS2-ERG* positive), *P*=5.60E−03; (Primary PCa, *TMPRSS2-ERG* negative) *vs* (Primary PCa, *TMPRSS2-ERG* positive), *P*=8.60E−03. (**C**) Data: Brase *et al*, 2011; *P*-values of group comparison for difference of mean PDE4D7 expression: (NAT *TMPRSS2-ERG* negative) *vs* (Primary PCa, *TMPRSS2-ERG* negative), *P*=7.80E−01; (NAT *TMPRSS2-ERG* negative) *vs* (Primary PCa, *TMPRSS2-ERG* positive), *P*=5.10E−07; (Primary PCa, *TMPRSS2-ERG* negative) *vs* (Primary PCa, *TMPRSS2-ERG* positive), *P*=3.80E−06. **P<0.01 and ***P<0.001.

To investigate this further, we set out to establish if there was any correlation between PDE4D7 expression and factors known to regulate initial tumorigenesis in the prostate. The *TMPRSS2-ERG* gene fusion has previously been reported as a clinical indicator for prostate cancer formation. Since its discovery, this prostate cancer-specific fusion event has been described in ∼50% of prostate cancer patients and has become a molecular hallmark of prostatic tumours (Tomlins *et al*, 2005). Given the status of *TMRSS2-ERG* as the most relevant genomic fusion event so far identified in prostate cancer, we tested the expression of PDE4D7 in 1106 patients with (Primary PCa, *TMPRSS2-ERG* positive; Figure 3) and without (Primary PCa, *TMPRSS2-ERG* negative; Figure 3) this gene fusion. Figure 3A–C shows the PDE4D7 expression across three exon array data sets (data sets Taylor *et al*, 2010; Brase *et al*, 2011; Boormans *et al*, 2013; Böttcher *et al*, 2015; Supplementary Table 1), which we picked for graphical illustration (all data sets where we had information on *TMRPSS2-ERG* rearrangement information available can be found in Supplementary Table 7). Intriguingly, we observed a significantly higher PDE4D7 expression in tumour samples that harboured the *TMPRSS2-ERG* gene fusion when compared with *TMPRSS2-ERG* negative samples or when compared with NAT (2-fold median increase, with some samples in excess of 30-fold upregulation; *P*-values of group-wise comparisons between *TMPRSS2-ERG* negative *vs* positive tumours: 3.33E−08; 8.60E−03; 3.80E−06, respectively). At the same time there was no significant expression difference observed between *TMPRSS2-ERG* gene fusion negative cancer samples and NAT (Figure 3A–C; Supplementary Table 7).

### PDE4D7 expression is positively correlated with low-grade *TMPRSS2-ERG*-positive prostate tumours

**Figure 4:**
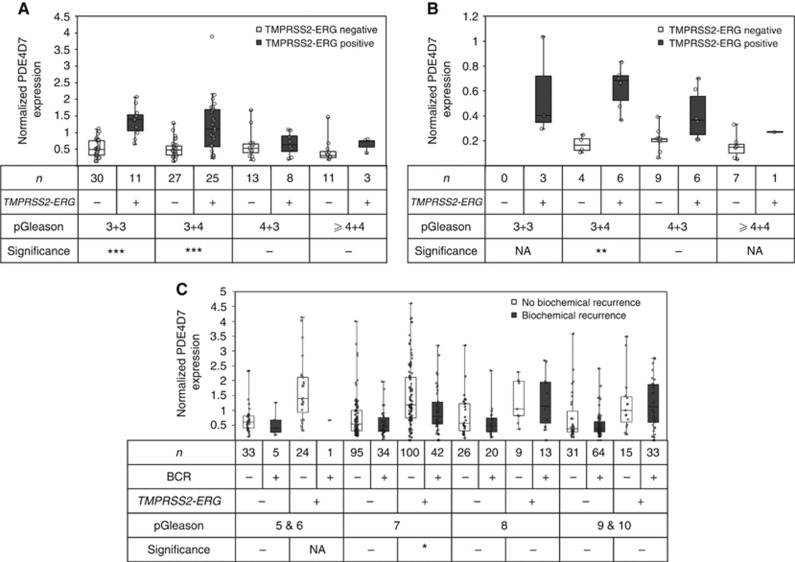
**Correlation of PDE4D7 expression to pathology gleason score.** (**A** and **B**) Box-and-Whisker plots of the normalised PDE4D7 transcript expression in various prostate cancer tissues. For all data sets, and all *P*-values see Supplementary Table 8 (data sets Taylor *et al*, 2010; Brase *et al*, 2011) and Supplementary Table 9 (data set Erho *et al*, 2013). For estimation of positive *TMPRSS2-ERG* fusion status see Materials and Methods. Patient cohorts were categorised according to their pGleason on histology as indicated. (**A**) Data Taylor *et al*, 2010; *P*-values of group comparison for difference of mean PDE4D7 expression: (pGleason 3+3 & 3+4 (*TMPRSS2-ERG* negative)) *vs* (pGleason 4+3 & ⩾4+4 (*TMPRSS2-ERG* negative)), *P*=4.80E−01; (pGleason 3+4 & 3+4 (*TMPRSS2-ERG* positive)) *vs* (pGleason 4+3 & ⩾4+4 (*TMPRSS2-ERG* positive)), *P*=2.40E−03; (**B**) Data Brase *et al*, 2011; *P*-values of group comparison for difference of mean PDE4D7 expression: (pGleason 3+3 & 3+4 (*TMPRSS2-ERG* negative)) *vs* (pGleason 4+3 & ⩾4+4 (*TMPRSS2-ERG* negative)), *P*=8.20E−01; (pGleason 3+4 & 3+4 (*TMPRSS2-ERG* positive)) *vs* (pGleason 4+3 & ⩾4+4 (*TMPRSS2-ERG* positive)), *P*=4.20E−02; (**C**) Data Erho *et al*, 2013; progression after primary treatment (i.e., surgery) is indicated as BCR (+) or absence (−) of BCR. *P*-values of group comparison for difference of mean PDE4D7 expression: (pGleason 7 (*TMPRSS2-ERG* negative), NP) *vs* (pGleason 7 (*TMPRSS2-ERG* negative, BCR)), *P*=1.10E−01; (pGleason 7 (*TMPRSS2-ERG* positive), NP) *vs* (pGleason 7 BCR (*TMPRSS2-ERG* positive), BCR), *P*=4.60 E−02.

Having discovered a strong correlation between *TMPRSS2-ERG* fusion and PDE4D7 expression, we then set out to ascertain if cancer aggressiveness is correlated with PDE4D7 expression. We compared the transcript levels of PDE4D7 against pathology-graded cancer samples utilising three exon array data sets (Taylor *et al*, 2010; Brase *et al*, 2011; J Schalken, Personal Communication; Supplementary Table 1), as well as the TCGA prostate adenocarcinoma RNA seq Data Set Prostate Cancer (Release September 2013). We categorised Gleason score (pGleason) into the following four groups of increasing grade: (1) pGleason 3+3, (2) pGleason 3+4, (3) pGleason 4+3, (4) pGleason ⩾4+4. A total of 264 patients were included in this stratification, and Supplementary Table 8 provides an overview of various group-wise comparisons of these different pGleason groups. Amazingly, a significant downregulation of PDE4D7 between low grade (pGleason ⩽3+4) *vs* high grade (pGleason ⩾4+3) tumours was only observed in patients possessing the *TMPRSS2-ERG* gene fusion (Figure 4A and B; Supplementary Table 8). The initial increase in PDE4D7 expression in low-grade prostate cancer is in keeping with our observations from Figure 3. It is significant that in *TMPRSS2-ERG*-positive tumour samples the expression of PDE4D7 is negatively correlated with increasing pGleason, highlighting the transient nature of PDE4D7 upregulation. This finding bears a striking resemblance to our previous observations in cell lines and xenografts (Henderson *et al*, 2014).

### PDE4D7 expression is correlated with clinical outcome in patients expressing the *TMPRSS2-ERG* gene fusion

To test our hypothesis that PDE4D7 expression can predict clinical outcome in patients with positive *TMPRSS2-ERG* gene rearrangement, we used an exon array data sets covering 527 eligible patient samples where longitudinal outcome data was available (Taylor *et al*, 2010; Boormans *et al*, 2013; Erho *et al*, 2013). The data allowed for prediction of BCR after primary treatment. The patients were grouped according to their *TMPRSS2-ERG* gene fusion status, as well as according to pGleason (5 and 6, 7, 8, and 9 and 10). We then compared the PDE4D7 expression in patient groups with *vs* without BCR during 5-years follow-up after primary treatment (Figure 4C and Supplementary Table 9). We could not detect a significant change in the expression of PDE4D7 in any of the *TMPRSS2-ERG*-negative pGleason groupings. However, for patient groups with positive *TMPRSS2-ERG* gene fusion status we found significant differential expression in the pGleason 7 group between no progression and BCR during follow-up, while this was not the case for the pGleason scores >7. Unfortunately, there is only a single patient sample in the pGleason 5 and 6 group with positive *TMPRSS2-ERG* status and progression to BCR so we could not calculate a *P*-value. However, this particular sample shows a very low PDE4D7 expression value compared with the samples in this pGleason group but without post-treatment progression (Figure 4C). We concluded from this that low PDE4D7 expression values in patient samples with low pGleason scores (6 and 7) are associated with an increased likelihood of biochemical failure after primary intervention.

**Figure 5:**
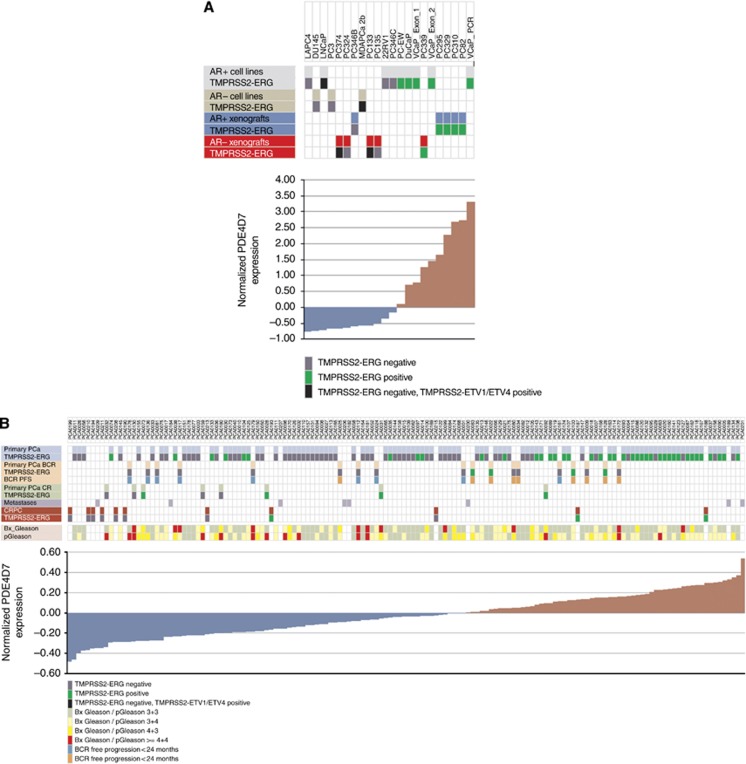
**Correlation of PDE4D7 expression in cancerous human prostate tissues to patient outcome.** A range of prostate Cancer Cell lines, xenografts, as well as patient prostate cancer tissues (data Taylor *et al*, 2010; Boormans *et al*, 2013; Böttcher *et al*, 2015; Supplementary Table 1) are ranked according to PDE4D7 expression in the respective cells or tissues. The normalised PDE4D7 expression value of each sample was adjusted by subtracting the mean of all expression values of the sample set. Details of cell lines, xenografts and patient samples can be found in Supplementary Table 4. (**A**) PDE4D7 expression in cell lines and xenograft tissues; (**B**) PDE4D7 expression in patient samples. For all samples its rank as well as the *TMPRSS2-ERG*, *−ETV1* or −*ETV4* fusion status is indicated. The BCR progression free survival (BCR PFS) after surgery (<24 months *vs* >24 months) is indicated. Further the biopsy Gleason score (Bx_Gleason) as well as the pathology Gleason (pGleason) is given. Samples are categorised into the following groups: AR+ cell lines—androgen-sensitive cell lines; AR− cell lines—androgen-insensitive cell lines; AR+ xenografts—androgen-sensitive xenografts; AR− xenografts—androgen-insensitive xenografts; CRPC—castration resistant prostate cancer; metastases—metastatic tumour; primary PCa—primary prostate cancer, no progression during follow-up; primary PCa BCR—primary prostate cancer, progression to BCR during follow-up; primary PCa CR—primary prostate cancer, progression to CR during follow-up.

A graphical representation of PDE4D7 expression in various cell and tissue types including AR negative/AR positive cell lines and xenografts, primary prostate cancer with and without progression to biochemical or CR, metastases and CRPC is shown in Figure 5A (cell lines and xenograft samples) and Figure 5B (patient samples; Supplementary Table 4). The samples are ordered based on their normalised PDE4D7 expression. For the cell lines, xenografts, primary tumours without progression and primary tumours with progression to BCR or CR, as well as CRPC tumours, the status of the *TMPRSS2-ERG* rearrangement is indicated. In general, the more aggressive type of samples are represented by low expression levels of PDE4D7, while less aggressive samples show elevated PDE4D7 expression. It is evident from the depicted cell lines and xenografts that the expression level of PDE4D7 is largely influenced by its *TMPRSS2-ERG* rearrangement status rather than its AR expression status, where AR positive cell lines without gene fusion show low PDE4D7 expression, while cell lines of the same category but positive gene translocation demonstrate high PDE4D7 expression levels (Figure 5A). It is also of importance to note that this effect seems to be very specific to the ERG translocation as cell lines or xenograft samples with ETV1 or ETV4 translocations do not show elevated PDE4D7 transcription (Figure 5A). Also, looking at the samples collected from patients without disease progression during follow-up reveals that those samples that were positively tested for *TMPRSS2-ERG* in general show increased expression of PDE4D7 (Figure 5B). This was also the case for primary tumour samples where patients progressed to either biochemical or CR as well as for CRPC. We further annotated for patients who experienced a biochemical relapse the time to PSA recurrence as two categories—relapse <24 months *vs* relapse >24 months after primary treatment. We observed a clear association between an increased PDE4D7 expression level and an elevated time to recurrence (*P*=1.72E−02; eight out of nine patients with normalised PDE4D7 expression >0 had a BCR recurrence event >24 months after primary therapy; Figure 5B). Furthermore, we noticed that from eight patients with clinical disease recurrence during follow-up seven patients showed normalised PDE4D7 expression values <0 (Figure 5B) while only in one patient tissue we could measure PDE4D7 expression values >0 (Figure 5B).

**Figure 6:**
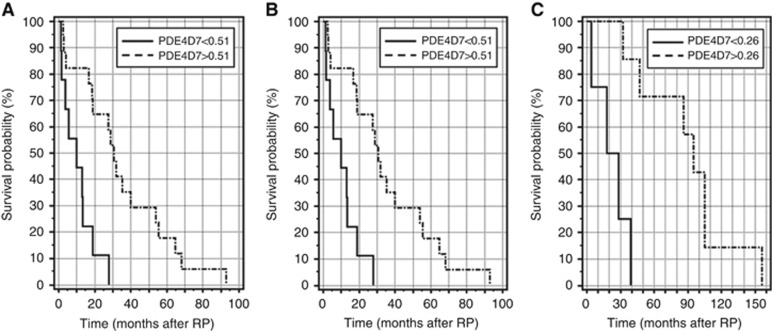
**Kaplan–Meier survival curves in men with biochemical recurrence after primary treatment.** (**A** and **B**) Using a cut-off of <0.51 (selected PDE4D7 expression level where the sum of the sensitivity and specificity in the ROC analysis reaches a maximum) for normalised PDE4D7 expression two patient cohorts can be separated with a median time to biochemical recurrence (BCR) after primary treatment of 9.9 and 9 months *vs* a median time to BCR of 30.6 and 50 months, respectively (HR=0.29; *P*=6.0E−04; HR=0.36; *P*=1.6E−03, respectively) (data: Taylor *et al*, 2010; Boormans *et al*, 2013; Böttcher *et al*, 2015; Supplementary Table 1). (**C**) Correlation of low expression of normalised PDE4D7 expression to time to BCR after primary treatment to time to clinical recurrence (data: Boormans *et al*, 2013). A cut-off as determined by ROC analysis of <0.26 for normalised PDE4D7 expression confirmed a fivefold increased risk (HR=0.2; *P*=2.0E−03) of reaching the endpoint of metastatic disease within a median of 18 months after surgery *vs* 95 months for PDE4D7 expression >0.26.

To further confirm this, we investigated the PDE4D7 expression in samples of patients that all underwent BCR during follow-up in one data set (Taylor *et al*, 2010). To segregate the patients into two survival groups, we applied a PDE4D7 expression value which was derived from a ROC analysis between patients who had BCR<24 months *vs* patients with BCR>24 months. We determined the unique point of PDE4D7 expression in the ROC curve where the sum of the sensitivity and the specificity becomes a maximum (i.e., <0.51) and used this factor for the Kaplan–Meier analysis. By this we could separate two patient cohorts (HR=0.29; *P*=6.0E−04) with a median time to BCR after primary treatment of <10 months *vs* a median time to BCR of >30 months (Figure 6A). When applying the same cut-off of <0.51 in an analysis of an independent data set (Boormans *et al*, 2013), we could verify this correlation to time to BCR after surgery (HR=0.36; *P*=1.6E−03) in this patient cohort with either a median time to BCR of <10 months, or a median time to recurrence >50 months (Figure 6B). The correlation of low PDE4D7 expression to time to BCR after primary treatment was further re-enforced in the second data set (Boormans *et al*, 2013), where time to CR demonstrated a fivefold increased risk of reaching the endpoint of metastatic disease within a median of 18 months after surgery when applying a cut-off <0.26 for PDE4D7 expression compared with a median time to CR of 95 months if PDE4D7 expression was >0.26 (HR=0.2; *P*=2.0E−03) (Figure 6C). This data strongly supports our hypothesis that low expression of PDE4D7 correlates with increased short-term biochemical reoccurrence, as well as manifestation of metastatic disease.

Most samples collected from CRPC patients demonstrated low PDE4D7 expression levels while again those samples that were positive for the *TMPRSS2-ERG* fusion gene were measured with increased PDE4D7 transcription (Figure 5B). Whether CRPC patients with positive gene fusion and PDE4D7 expression >0 will survive longer compared with patients with negative *TMRPSS2-ERG* fusion and PDE expression <0 is a very interesting subject for further research.

## Discussion

Analysis of data from large scale genome sequencing projects like TCGA has uncovered a potential role of the PDE4D gene in various types of cancer (Zack *et al*, 2013). Indeed, loss of *PDE4D* was noted as one of the 10 most relevant gene deletion events in 1 study cohort (Baca *et al*, 2013). Although *PDE4D* copy number and, to a lesser degree, mutational status correlates with cancer incidence the role of *PDE4D* isoform expression has not been studied in a clinical context.

Recent studies have implicated individual PDE4D transcripts in the development of prostate cancer (Rahrmann *et al*, 2009; Henderson *et al*, 2014). Specifically, we reported for the first time the downregulation of PDE4D7 in hormone-refractory prostate disease represented by a wide range of both cellular and xenograft models (Henderson *et al*, 2014). Here, we set out to discern whether the differential regulation of PDE4D7 could be verified in human tissue samples collected from primary, as well as metastatic and castration resistant tumours. Encouragingly, across multiple data sets we were able to detect a clear and significant downregulation of PDE4D7 transcript abundance correlating with increasing prostate disease aggressiveness (as assessed by increasing pGleason score and disease stage).

We previously demonstrated that selective knockdown of PDE4D7 expression in androgen-sensitive cell line models led to a more aggressive phenotype, while its overexpression in CRPC cells had the opposite effect (Henderson *et al*, 2014). The precise details of the cAMP signalling pathways regulated by PDE4D7 during the development of aggressive prostate cancer remain to be uncovered and are subject to future research. However, we would like to propose that PDE4D7 has a contributing role in initial prostate cancer cell states rather than having a ‘passenger effect' occurring as a consequence of the molecular changes induced by other factors. To understand the baseline for PDE4D7 expression, and thereby contextualise the differential regulation of this particular PDE isoform during prostate cancer development and progression, we examined its expression status in normal prostate tissue compared with primary and advanced prostate cancers. Notably, the expression of the PDE4D7 transcript was significantly lower in normal, as well as tissue of benign origin compared with low-grade prostate tumours. This leads us to propose a model, where PDE4D7 expression becomes upregulated in primary disease. This, perhaps, reflects an attempt by cells to counteract the proliferative phenotype, before the failure/overcoming of this response leads to PDE4D7 downregulation, which characterises the more aggressive prostate tumours. Thus PDE4D7 appears to be functionally involved in the primary development of prostatic tumours. However, our data suggests that future cellular and molecular studies could usefully be directed to ascertain whether the initial upregulation of PDE4D7 is intimately involved in the initial stage of prostate tumorigenesis.

Interestingly, we uncover here a novel link between AR signalling and PDE4D7 expression by correlating the incidence of *TMPRSS2-ERG* gene fusion and PDE4D7 transcript levels. The *TMPRSS2-ERG* gene fusion between the prostate specific serine protease *TMPRSS2* and the ETS transcription factor family member ERG was first detected in 2005 by a statistical outlier approach (Tomlins *et al*, 2005). Subsequently, this gene fusion has been shown to be present in ∼50% of prostate cancer patients and is, consequently, one of the most prominent genomic fusion events reported in prostate cancer (Kumar-Sinha *et al*, 2008). This translocation results in androgen-regulated ERG expression such that the androgen-responsive promoter of TMPRSS2 now drives *TMPRSS2-ERG* expression, resulting in an upregulation in both the expression and activity of the transcription factor, ERG (Tomlins *et al*, 2005). However, despite numerous studies the clinical implications and functional consequences of the genomic fusion remain to be fully understood (Petrovics *et al*, 2005; Mosquera *et al*, 2007; Saramäki *et al*, 2008; Hermans *et al*, 2009). Here, we uncover a remarkably significant difference in PDE4D7 expression between *TMPRSS2-ERG*-negative and *TMPRSS2-ERG*-positive tumour samples. Indeed, when stratified by *TMPRSS2-ERG* incidence it is clear that PDE4D7 is most significantly upregulated in low-grade *TMPRSS2-ERG*-positive tumours. This raises the possibility that PDE4D7 expression may be directly or indirectly regulated by the aberrant transcriptional activity of the *TMPRSS2-ERG* fusion protein. Inspection of the *PDE4D* gene reveals several putative binding sites for ERG, one within the promoter region of PDE4D7 (Materials and Methods). It would therefore seem logical that if PDE4D7 is regulated by ERG transcription, an increase in the expression of the androgen-regulated TMPRSS2-ERG factor would lead to a concurrent androgen-driven increase in PDE4D7 expression.

To date, most newly detected prostate cancer cases are clinically classified low-risk diseases (Bangma and Roobol, 2012). It is crucial to understand the natural history of these tumours as it is under considerable debate whether and to what extent low-risk Gleason 6 tumours are able to progress to higher grade tumours leading to metastatic spread or even cancer-specific death (Whittemore *et al*, 1991; Sowalsky *et al*, 2013). Interestingly, our data may indicate that reduced expression of PDE4D7 in low to intermediate Gleason tumours is correlated to progression after primary treatment. Although initially positively correlated with tumour development, the expression of PDE4D7 actually appears to be protective against further disease progression, which is in line with the data previously obtained regarding the cellular functioning of PDE4D7 (Henderson *et al*, 2014).

As new strategies for targeted pharmacological manipulation of specific PDE4D transcripts become available then PDE4D7 likely provides a promising future target in the treatment of primary and/or advanced prostate cancer. Our data indicate that during tumour progression the risk of fast recurrence to clinical endpoints like biochemical or clinical disease is correlated to the level of PDE4D7 expression in the primary tumour. Consequently, patients with a low expression level of PDE4D7 in their primary cancers after surgical resection may very well be candidates for immediate adjuvant treatment like radiotherapy and/or androgen ablation. Furthermore, the manipulation of PDE4D7 suggests a strategy to selectively treat *TMPRSS2-ERG* fusion-positive prostate cancers. However, the success of such strategy may depend on the stratification into molecular sub-types according to the status of the *TMPRSS2-ERG* gene translocation.

The data presented here demonstrates the relevance of PDE4D7 as a potential biomarker for more accurate prostate cancer diagnostics. In particular, we have demonstrated the potential role of this specific splice variant of the *PDE4D* gene for prognosis of aggressive prostate cancer in the molecular sub-type of *TMPRSS2-ERG*-positive prostate tumours as well as its role as a putative target gene for therapy of primary *vs* late-stage, hormone-refractory disease.
