# Supplementary material for: Human phosphodiesterase 4D7 (PDE4D7) expression is increased in TMPRSS2-ERG-positive primary prostate cancer and independently adds to a reduced risk of post-surgical disease progression
Source: Br J Cancer. 2015 Nov 17;113(10):1502–11. doi: 10.1038/bjc.2015.335 (PMC4815894; doi:10.1038/bjc.2015.335)
Supplement: Supplementary Information [file bjc2015335x1.docx]

**Supplemental Material**

**Supplemental Table Legends**

**Supplementary Table 1**: Summary overview of patient samples and publically available data sets used in the study. Source of the tissues or data is indicated as well as the used technology platform. NAT – Normal, Adjacent Tissue; BL – Benign Lesions; BPH – Benign Hyperplasia; Primary PCa, NP – primary prostate cancer, non-progressive; Primary PCa, BCR – primary prostate cancer, biochemical recurrence; Primary PCa, CR – primary prostate cancer, clinical recurrence; Metastases – metastatic prostate cancer samples; CRPC – Castration Resistant Prostate Cancer; the number of samples per patient group and data set are indicated as well as the status of TMPRSS2-ERG gene rearrangement (- not available; + available). The columns ‘Figure’ and ‘Table’ indicate which data set was used to generate Figures and Tables.

**Supplementary Table 2:** Summary overview of two patient cohort characteristics used in this study for qPCR expression analysis of PDE4D7 (data sets #1, Origene Inc, USA; and #2, EMC Rotterdam, The Netherlands in Supplementary Table 1).

**Supplementary Table 3:** Summary overview of qPCR primers and probes for PDE4D7 as well as the used reference genes are given.

**Supplementary Table 4:** Summary overview of cell lines, xenograft samples and patient samples used in Figure 5A and 5B. The following cell line and xenograft data was derived from Boormans *et al*, 2013: LNCaP, PC346c, LAPC4, 22RV1, MDAPCa2B, VCaP_Exon_1, PC82, PC133, PC135, PC295, PC310, PC324, PC329, PC339, PC346B, PC374, PCEW; the following cell line data was derived from Taylor *et al*, 2010: DU145, PC3, VCaP_Exon_2; the sample VCaP_PCR was in house culture VCaP cells and expression of PDE4D7 was performed by qPCR as described.

**Supplementary Table 5. Summary overview of PDE4D7 expression tested in patient cohorts with progressive disease.** Disease stages designated as in Figure 1A with the addition of Primary PCa, NP – primary prostate cancer, no disease progression during follow-up (data sets: 3, 4 and 6 as described in Supplementary Table 1). The number of patient samples per group is indicated. The p-values for the significance of the differential expression of normalized PDE4D7 expression (methods) are indicated. The ‘AUC’ represents the Area Under the Curve of the ROC (receiver operating characteristics) curve. The ‘95% CI’ as well as the p-value (confidence interval) for the AUC is given.

**Supplementary Table 6. Summary Overview of PDE4D7 Expression in Normal Adjacent Tissue vs. Primary Prostate Cancer.** Disease stages designated as in Figure 1A. The number of patient samples per group is indicated. The p-values for the significance of the differential expression of normalized PDE4D7 expression (methods) are indicated.

**Supplementary Table 7.** **Correlation of PDE4D7 Expression in Normal and Cancerous Human Prostate Tissues to the TMPRSS2-ERG Gene Fusion Status**. Disease stages designated as in Figure 1A. The number of patient samples per group is indicated. Positive *TMPRSS2-ERG* fusion status was estimated by transformation to robust z scores. Subsequently, a threshold of >3 was applied to define samples with positive fusion events. The p-values for the significance of the differential expression of normalized PDE4D7 expression (methods) are indicated.

**Supplementary Table 8. Correlation of PDE4D7 Expression in Cancerous Human Prostate Tissues to Pathology Gleason Depending on the Status of TMPRSS2-ERG Genome Rearrangement.** Disease stages designated as in Figure 1A. The number of patient samples per group is indicated. The pGleason categories used to create pGleason groups for comparison of PDE4D7 expression are given. The p-values for the significance of the differential expression of normalized PDE4D7 expression (methods) are indicated.

**Supplementary Table 9. Correlation of PDE4D7 Expression in Cancerous Human Prostate Tissues to Clinical Outcome Depending on the Status of TMPRSS2-ERG Genome Rearrangement.** Disease stages designated as in Figure 1A. The number of patient samples per group is indicated. Positive *TMPRSS2-ERG* fusion status was estimated by transformation to robust z scores. Subsequently, a threshold of >3 was applied to define samples with positive fusion events. The 5-year progression status of the patient group is indicated (NP - no progression during follow-up; BCR – biochemical recurrence during follow-up). The pGleason categories used to create pGleason groups for comparison of PDE4D7 expression are given. The p-values for the significance of the differential expression of normalized PDE4D7 expression (methods) are indicated.
